# Antimicrobial Efficacy of Glass Ionomer Cement in Incorporation with Biogenic *Zingiber officinale* Capped Silver-Nanobiotic, Chlorhexidine Diacetate and Lyophilized Miswak

**DOI:** 10.3390/molecules27020528

**Published:** 2022-01-14

**Authors:** Amal Adnan Ashour, Sakeenabi Basha, Nayef H. Felemban, Enas T. Enan, Amal Ahmed Alyamani, Sanaa M. F. Gad El-Rab

**Affiliations:** 1 Department of Oral and Maxillofacial Surgery and Diagnostic Sciences, Oral Pathology Division, Faculty of Dentistry, Taif University, Taif 26571, Saudi Arabia; a.a.ashour@tudent.edu.sa; 2 Department of Preventive and Community Dentistry, Faculty of Dentistry, Taif University, Taif 26571, Saudi Arabia; sakeena@tudent.edu.sa; 3 Preventive Dentistry Department, Faculty of Dentistry, Taif University, Taif 26571, Saudi Arabia; nfelemban@tudent.edu.sa; 4 Department of Dental Biomaterials, Faculty of Dentistry, Mansoura University, Mansoura 35511, Egypt; enasenan275@mans.edu.eg; 5 Department of Biotechnology, Faculty of Science, Taif University, Taif 21974, Saudi Arabia; a.yamani@tu.edu.sa; 6 Department of Botany and Microbiology, Faculty of Science, Assiut University, Assiut 71516, Egypt

**Keywords:** GIC, ZOE-AgNPs, miswak, chlorhexidine, antimicrobial efficacy

## Abstract

In the present study, *Zingiber officinale* is used for the synthesis of *Zingiber officinale* capped silver nanoparticles (ZOE-AgNPs) and compares the antimicrobial efficacy and compressive strength of conventional glass ionomer cement (GIC) combined with ZOE-AgNPs, lyophilized miswak, and chlorhexidine diacetate (CHX) against oral microbes. Five groups of the disc-shaped GIC specimens were prepared. Group A: lyophilized miswak and GIC combination, Group B: ZOE-AgNPs and GIC combinations, Group C: CHX and GIC combination, Group D: ZOE-AgNPs + CHX + GIC; Group E: Conventional GIC. Results confirmed the successful formation of ZOE-AgNPs that was monitored by UV-Vis sharp absorption spectra at 415 nm. The X-ray diffractometer (XRD) and transmission electron microscope (TEM) results revealed the formation of ZOE-AgNPs with a mean size 10.5–14.12 nm. The peaks of the Fourier transform infrared spectroscopy (FTIR) were appearing the involvement of ZOE components onto the surface of ZOE-AgNPs which played as bioreducing, and stabilizing agents. At a 24-h, one-week and three-week intervals, Group D showed the significantly highest mean inhibitory zones compared to Group A, Group B, and Group C. At microbe-level comparison, *Streptococcus mutans* and *Staphylococcus aureus* were inhibited significantly by all the specimens tested except group E when compared to *Candida albicans*. Group D specimens showed slightly higher (45.8 ± 5.4) mean compressive strength in comparison with other groups. The combination of GIC with ZOE-AgNPs and chlorhexidine together enhanced its antimicrobial efficacy and compressive strength compared to GIC with ZOE-AgNPs or lyophilized miswak or chlorhexidine combination alone. The present study revealed that The combination of GIC with active components of ZOE-AgNPs and chlorhexidine paves the way to lead its effective nano-dental materials applications.

## 1. Introduction

**Scheme 1:**
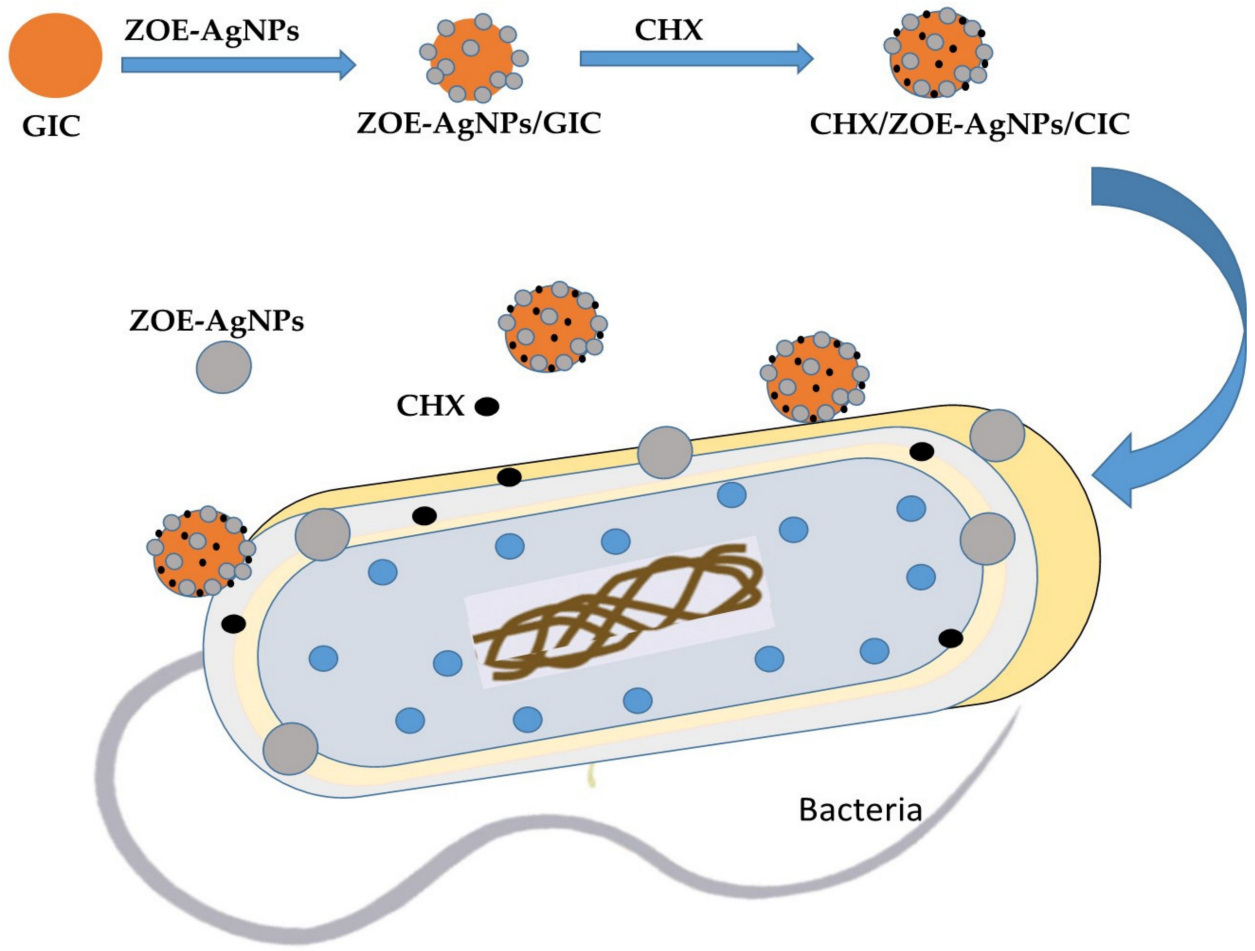
Schema of synthetic diagram for ZOE-AgNPs/CHX/GIC and their synergistic antimicrobial efficacy.

Glass ionomer cement (GIC) is widely accepted and is the most commonly used acid-based cement in the restoration of carious lesions due to its distinctive properties like chemical adhesion to the tooth structure, good biocompatibility, fluoride release, and reduced thermal expansion [1]. The antibacterial efficacy of GIC is attributed to its fluoride release property; however, it is not effective enough to inhibit microbial growth leading to secondary caries beneath the GIC restoration [2,3]. Along with low antimicrobial efficacy, GIC also exhibits high dissolution in water sorption, low wear-resistance, and low fracture toughness leading to restoration failures [3]. To overcome these limitations, various antimicrobial agents were incorporated into GIC to enhance its antimicrobial efficacy without affecting its mechanical properties [4,5,6,7]. Inorganic antimicrobial agents such as silver nanoparticles (AgNPs) offer long term antibacterial activity, high surface to volume ratio, low bacterial resistance, high thermal stability, and low volatility [8,9]. Due to their nano size, they can penetrate the microbial cell wall/membranes leading to cell death by damaging the microbial DNA [10]. Biosynthesized AgNPs have enhanced antimicrobial efficacy due to the added medicinal properties of plant extract used to synthesize the nanoparticles [11,12]. Previous studies have shown the incorporation of AgNPs enhanced antimicrobial efficacy and mechanical properties of conventional GIC without any cytotoxic effects on pulpal cells [13,14,15,16]. Miswak (*Salvadora persica*) is one of the most commonly used tooth cleaning tools since ancient times in many parts of the world. It is used as a toothbrush stick for treatment of gum inflammation and in oral hygiene. Extract of miswak possesses different antimicrobial and antifungal properties due to the presence of chlorides, trimethylamine, fluoride, silica, saponins, sulfur, flavonoids, and phenols [17]. Also, its antimicrobial efficacy is related to its b-sitosterol, and m-anisi acid [18]. Previous studies have shown significant antimicrobial efficacy of miswak extract against cariogenic microbes when is used alone and combined with conventional GIC [19,20]. Chlorhexidine gluconate is a gold standard antimicrobial agent, and previous in-vitro and in-vivo studies showed the combination of GIC with chlorhexidine enhanced its antimicrobial efficacy [6,21,22,23]. The study by Charannya et al. [24] showed the combination of AgNPs with 2% chlorhexidine enhanced its antimicrobial efficacy against *K. pneumonia, E. faecalis*, and *Candida albicans* (*C. albicans*) when compared to each of those solutions used individually. However, the past scientific literature did not offer any publication investigating the effect of the combination of AgNPs and chlorhexidine together in GIC. Hence, the present study aims to compare the antimicrobial efficacy and compressive strength of conventional GIC combined with biosynthesized AgNPs, miswak extract, or chlorhexidine and a combination of AgNPs and chlorhexidine together with GIC (Scheme 1) against *Streptococcus mutans* (*S. mutans*)*,* *Staphylococcus aureus (S. aureus),* and *C. albicans* *because* oral microorganisms like *S. mutans*, *S. aureus* and *C. albicans* with other microbes forms dental plaque [25].

## 2. Results and Discussion

### 2.1. Characterization of ZOE-AgNPS

**Figure 1:**
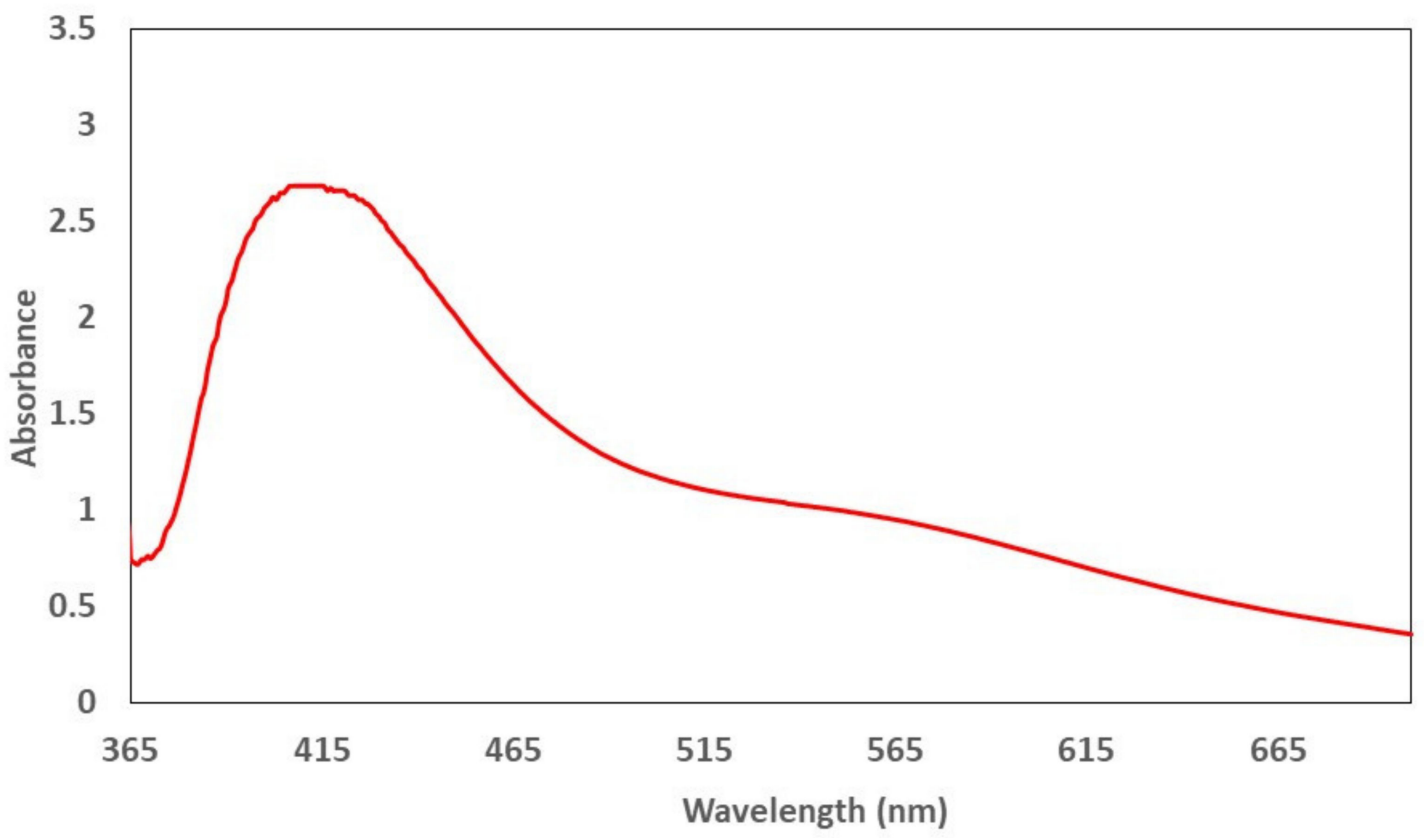
UV-Vis spectral range showing a peak of ZOE-AgNPs at 415 nm.

The UV-Vis spectrophotometric analysis peak displayed at 415 nm for ZOE-AgNPs (Figure 1). The colorless solution of AgNO_3_ turned into yellow in the presence ZOE and then to dark brown during the reaction. The color modification in the solution mixture suggested ZOE-AgNPs formation. Enan et al., studied the effect of leaf extract on the formation of AgNPs. The results obtained by them were very similar to what we found [26].

**Figure 2:**
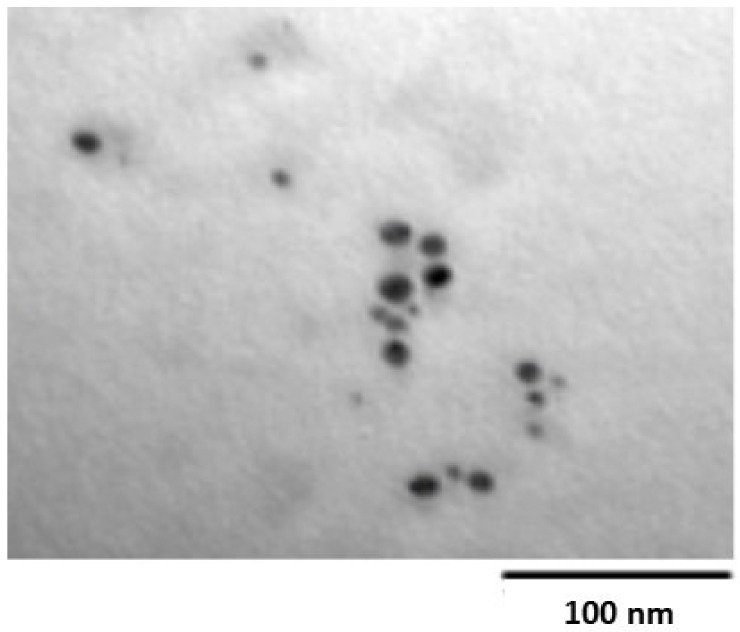
TEM image ZOE-AgNPs.

TEM image of the ZOE-AgNPs (Figure 2) displayed a spherical shape with average size from 10.5 to 14.12 nm. Our finding results were almost in agreement with previous findings [27].

**Figure 3:**
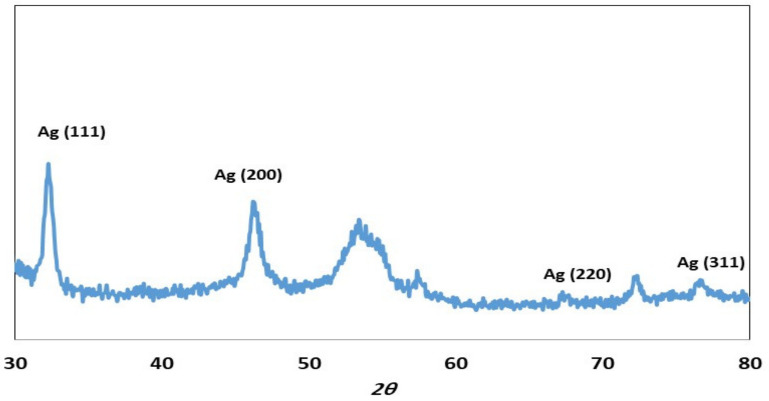
XRD pattern of AgNPs.

The XRD analysis is commonly used to display the crystal structure of the synthesized ZOE-AgNPs. Figure 3 shows the XRD pattern of ZOE-AgNPs synthesized by using ZOE. XRD pattern reveals the 2ϴ peaks obtained at 32.26°, 46.24°, 67. 24°, and 76.72° indexed with (111), (200), (220), and (311) planes, respectively, which confirmed the formation of ZOE-AgNPs with face-centered cubic structure of pure silver metal that was exhibited by all reflectance. Other peaks at 2θ values in ZOE-AgNPs pattern can be ascribed to the residues of the organic content of the ZOE. These peaks reveal the crystallization of some plant metabolite moieties on the surface of the ZOE-AgNPs, which is in agreement with Halawani et al.’s results [28].

**Figure 4:**
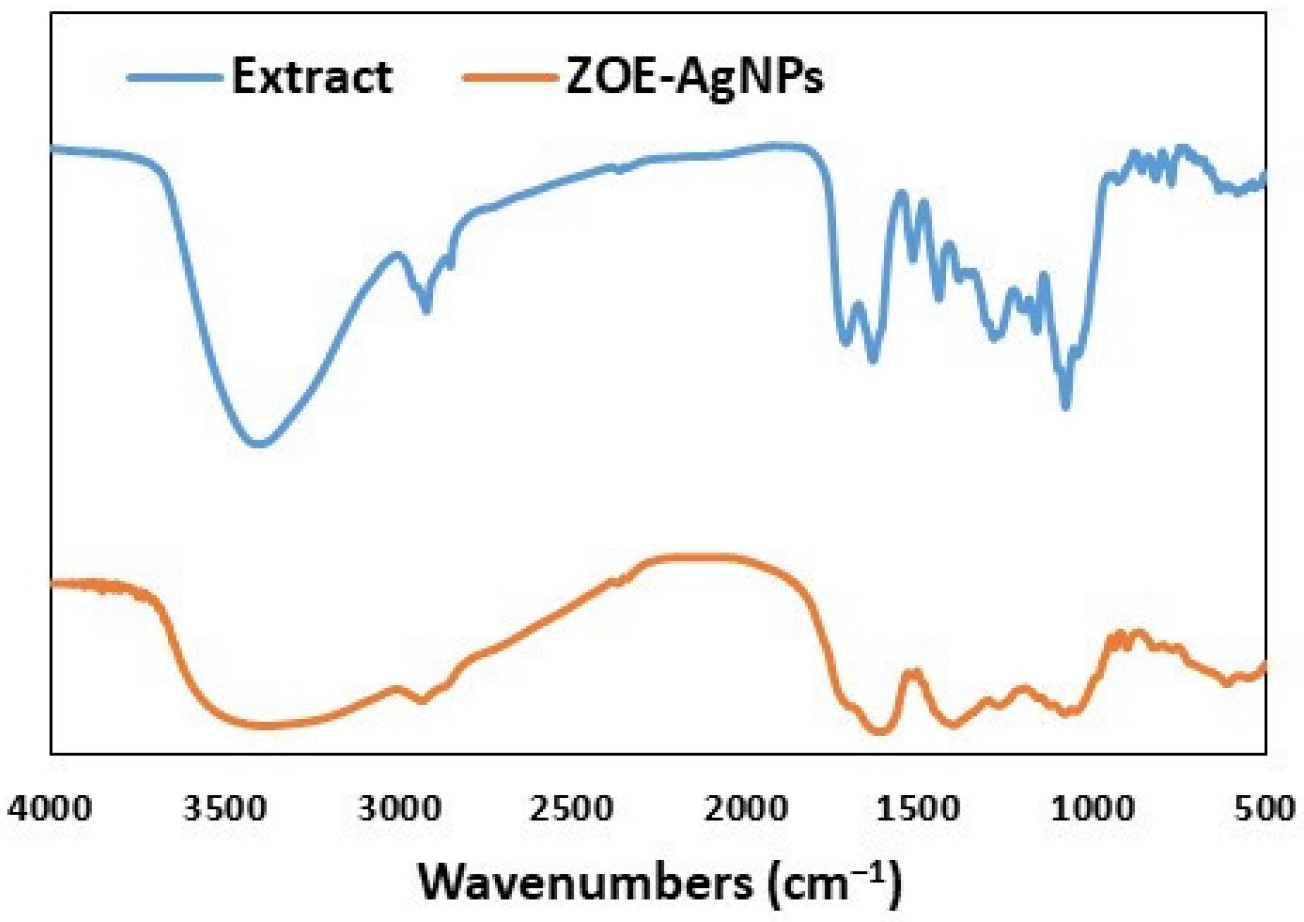
FT-IR spectra of AgNPs synthesized with ZOE.

The FTIR of ZOE-AgNPs was displayed as shown in Figure 4. The absorption peak at 3395 cm^−1^ represents the hydroxyl (OH) group, and alkyl CH stretch was recorded at 2917.89 cm^−1^. A peak at 1631.6 cm^−1^ can be attributed to the stretching vibrations of –COC (alkane). Sharp peak at 1075.34 cm^−1^, represents stretching vibrations of –COO. These peaks are present both in ZOE-AgNPs and ZOE. The bands at 916 and other peaks at 3395.34, 2917.89, 1604.55, 1075.34 cm^−1^, indicate the presence of various compounds such as phenolic compounds, carbohydrate, alkanoids, flavonoids and alkaloids, the active compounds of ZOE, which acts as the reducing and stabilizing agents [29].

### 2.2. Charcterization of Miswak

Miswak FTIR analysis and its corresponding functional groups related to benzyl isothiocyanate, alkaloids (salvadorine), sulfur, and benzyl cyanates (Data not shown) are all responsible for the growth inhibition of bacteria and fungus strains [30].

### 2.3. Antimicrobial Activity

Glass ionomer cement is the most commonly used restorative material due to its biocompatibility with the tooth structure [1]. The present study was the first of a kind that tested antimicrobial efficacy of GIC combined with miswak, ZOE-AgNPs, and chlorhexidine against *Streptococcus mutans,* *Staphylococcus aureus,* and *Candida albicans*. These microorganisms were selected in the present study due to their association with dental caries [31,32,33].

Table 1 shows the mean inhibitory zones of tested specimens. The mean inhibitory zones for all tested microbes were significantly high for Group D with GIC, AgNPs and chlorhexidine, Group A with GIC and miswak, Group B with GIC and AgNPs, and Group C with GIC and chlorhexidine combinations than Group E with GIC alone at a 24-h interval. At one week and three weeks intervals, Group D showed significantly higher mean inhibitory zones compared to Group A, Group B, and Group C.

**Figure 5:**
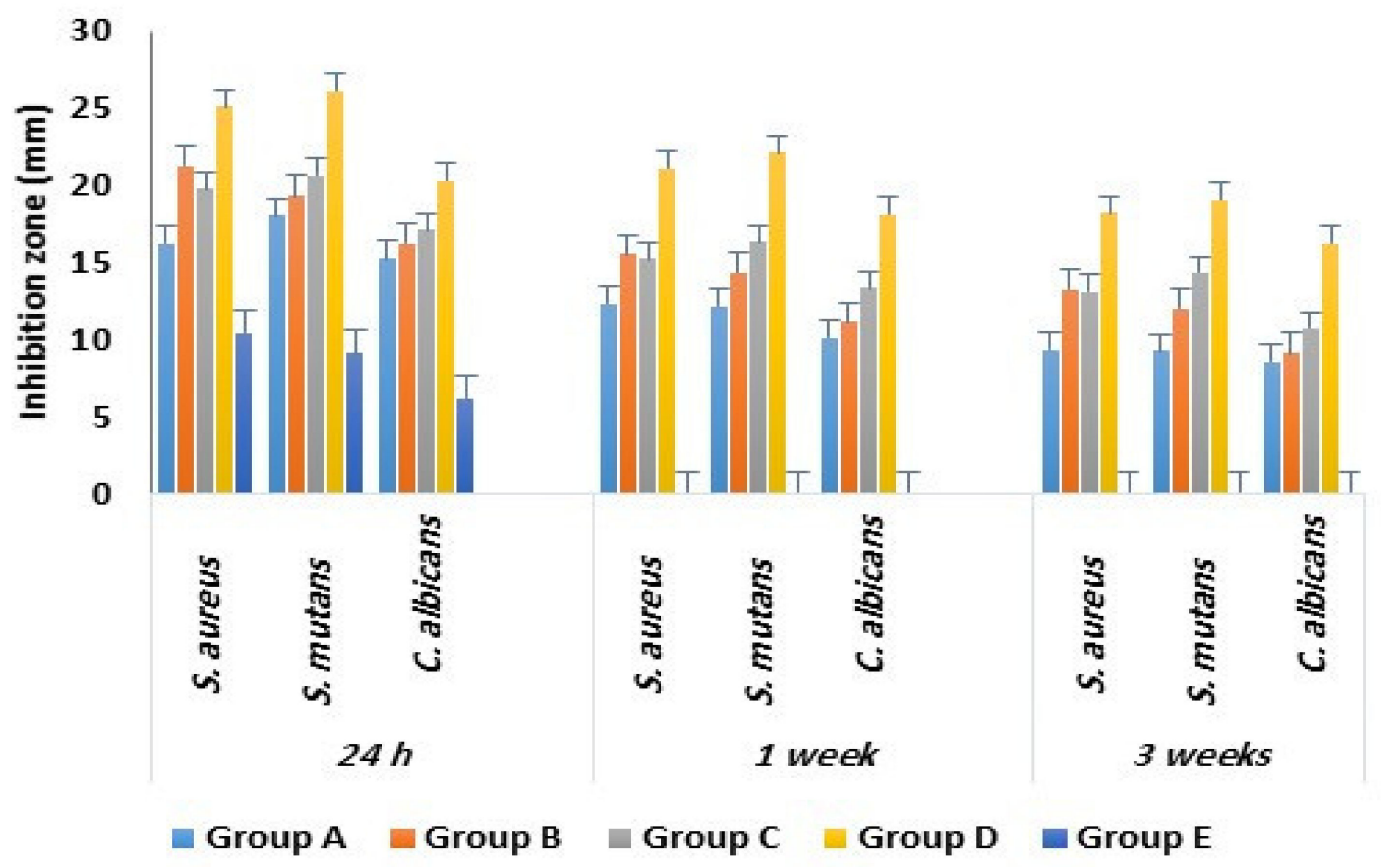
Antimicrobial efficacy of tested GIC specimens at different time intervals. GIC—Glass ionomer cement, Group A—GIC + lyophilized miswak, Group B—GIC + ZOE-AgNPS, Group C—GIC + chlorhexidine diacetate, Group D—GIC + ZOE-AgNPs + Chlorhexidine, Group E—control (glass ionomer alone).

At a microbe-level comparison, *Streptococcus mutan,* and *Staphylococcus aureus* were inhibited significantly by all the specimens tested compared to *Candida Albicans* (Figure 5).

Previously, authors have studied the antimicrobial efficacy of GIC combined with miswak, AgNPs, and chlorhexidine alone and showed that a combination of GIC with antimicrobial agents enhanced its antimicrobial efficacy without altering its mechanical properties [6,13,14,15,16,19,20,21,22,23,24]. However, in the present study, and for the first time, as shown in Table 2, GIC was combined with ZOE-AgNPs and chlorhexidine, and the result showed significantly higher antimicrobial efficacy against tested microbes compared to GIC with a single antimicrobial agent as well as conventional GIC without any combinations. Previous studies showed that chlorhexidine encapsulated in silica nanoparticles enhanced its antimicrobial efficacy [34,35]. The study by Charannya et al. [24] showed that a combination of AgNPs with 2% chlorhexidine enhanced its antimicrobial efficacy when compared to each of those solutions used individually.

The present study showed that an addition of lyophilized miswak to GIC enhanced its antimicrobial efficacy. This is in agreement with previous in vitro and in vivo studies which showed that the addition of miswak extract enhanced the antimicrobial efficacy of conventional GIC [19,20]. According to the present study result, the mean inhibitory zone was significantly higher for ZOE-AgNPs and chlorhexidine combined GIC at one week and three week intervals compared to lyophilized miswak combined GIC, ZOE-AgNPs combined, GIC, and chlorhexidine combined GIC. This might be due to the substantive property of ZOE-AgNPs and chlorhexidine together enhancing its antimicrobial efficacy at one- and three-week intervals [9,23].

The present study result showed that GIC combined with antimicrobial agents showed superior antibacterial activity against *Streptococcus mutans* and *Staphylococcus aureus* than antifungal activity against *Candida albicans.* This result is in line with previous studies [9,19,25], which showed different zones of inhibition against different oral microbes due to variance in the membrane permeability of the studied microorganism [37]. However, a combination of GIC with chlorhexidine and ZOE-AgNPs together enhanced its antifungal activity compared to GIC with chlorhexidine or ZOE-AgNPs alone. This result is in line with a previous study by Charannya et al., [24] who showed that a combination of nano-silver with chlorhexidine enhanced its antifungal activity.

### 2.4. Compressive Strength Measurement

Group D specimens’ slightly higher (45.8 ± 5.4) mean compressive strength compared to Group A (43.2 ± 3.1), Group B (44.7 ± 4.8), Group C (43.9 ± 3.6), and Group E (43.4 ± 3.2) (Table 3).

The present study result showed that a combination of conventional GIC with AgNPs and chlorhexidine together and AgNPs alone showed slightly higher mean compressive strength compared to conventional and miswak extract combined GIC, however, the difference was not statistically significant. This is in agreement with the previous studies [6,13,14] which showed the addition of silver nanoparticles enhanced mechanical properties of conventional GIC including its compressive strength.

The study limitation included it’s in vitro type, as it was not possible to simulate all the oral conditions in the lab setting. Future in vivo studies can confirm the result of the present study.

## 3. Materials and Methods

### 3.1. Study Design, Microbial Strains Used, and Ethical Approval

An in-vitro study was conducted at the Department of Microbiology and University Dental Hospital, Taif University, KSA. Ethical clearance was obtained from the Institutional review board, Taif University (Ethical clearance number-41-1107-00152). To evaluate antimicrobial efficacy, one strain of *candida* and two bacterial strains were used in this study *(Candida albican, Streptococcus mutans,* and *Staphylococcus aureus*) [38].

Materials used:

(a) The roots of miswak/*Salvadora persica* (SP) that were at least six months old, purchased from a local market, Taif City, KSA.
(b) The *Zingiber officinale* plant, purchased from a local market, Taif City, KSA.
(c) Conventional GIC (GC Fuji IX, Tokyo, Japan).
(d) Chlorhexidine diacetate powder (RM1659-25G, HiMedia Laboratories, Mumbai, India).

### 3.2. Preparation of Test Specimens

Miswak (*Salvadora persica*) extract preparation: The miswak roots were ground in a blender (Tomado Chopper, model No. TM-1273,). The 10 g of grounded miswak powder was mixed with 100 mL of deionized water in a sterilized screw-capped bottle. The mixture was kept in the refrigerator for 48 h below 4 °C and then centrifuged at 313 rpm for 10 min. The 100% aqueous miswak extract was obtained by filtering the supernatant using 0.45 mm filter paper, lyophilized and stored at −20 °C till further use.

### 3.3. Biosynthesis of Silver Nanoparticles

Silver nanoparticles were biosynthesized using *Zingiber officinale* extract (ZOE, 10%) [39] with 1 mM AgNO_3_ and characterized using transmission electron microscopy (TEM), Fourier transform infrared analysis (FT-IR), UV-visible spectrum, and X-ray diffraction analysis [40,41,42].

### 3.4. Preparation of GIC Combination Specimens

The disc-shaped GIC specimens were prepared using split Teflon moulds (3 mm height and 6 mm diameter). The modified GIC specimens were portioned and mixed according to the manufacturer’s instructions, placed in the molds, allowed to sit for 20 min, and then were removed from the mold and were sterilized using ultraviolet radiation for 30 min. A total of 150 specimens were divided into five groups. Then, each of the main groups was tested for antimicrobial efficacy against tested bacteria at three-time intervals of one day, one week, and three weeks. Each of the main groups was performed in triplicates (Table 4).

### 3.5. Determination of Antimicrobial Activity

A disc diffusion method was used to determine the antimicrobial activity [43]. The *Streptococcus mutan,* and *Staphylococcus aureus* were activated in Mueller-Hinton broth and *Candida albican* in Sabouraud dextrose. The 0.1 mL (1.5 × 10^8^) of activated bacteria and *Candida albicans* were inoculated in Mueller-Hinton agar medium and Sabouraud dextrose agar media, respectively. The GIC specimen discs were placed in inoculated medium. The inoculated plates were incubated at 37 °C for 24 h. The diameter of the inhibition zone in mm (DIZ) of the tested microbes determined the antimicrobial activity specimen groups. All tests were performed in triplets. The antimicrobial efficacy was tested at three intervals: 24-h, one week, and three weeks. The GIC specimens were stored in distilled water at 37 °C during the testing intervals.

### 3.6. Compressive Strength Measurement

To test the compressive strength (CS), cylindrical specimens (6 mm height and 4 mm diameter) were prepared using a Teflon mold. Before removal from the Teflon mold, the specimens were allowed to set at room temperature for 20 min. The CS of set cement was measured using a Material Test System (810 MTS Co., Minneapolis, MN, USA) at a crosshead speed of 0.5 mm/min, after 24 h of mixing. For each specimen group, ten specimens were tested. The maximum recorded force at the fracture was measured and CS (N/mm^2^) was determined using the following Equation (1) [29].

CS = 4P/πd^2^ (1)

where P represents the failure load, and d represents the diameter of the specimen.

### 3.7. Statistical Analysis

Mean difference was tested using One-way Analysis of Variance (ANOVA) followed by Tukey’s Post Hoc using the Statistical Package for Social Science version 17 (SPSS Inc., Chicago, IL, USA). All statistical tests were two-sided, and the significance level was set at *p* < 0.05.

## 4. Conclusions

Within the limitation of the present study, the ZOE-AgNPs was synthesized using ZOE and characterized using means of innumerable analytical methods. It can be concluded that the combination of GIC with synthesized ZOE-AgNPs and chlorhexidine together enhanced its antimicrobial efficacy and compressive strength compared to GIC with ZOE-AgNPs, miswak extract or chlorhexidine combination alone. Antimicrobial efficacy was higher against *Streptococcus mutans* and *Staphylococcus aureus* in comparison to *Candida albicans.*

## Figures and Tables

**Table 1:** Antimicrobial efficacy of tested GIC specimens at different time intervals.

Study Groups with Tested Microbes	Inhibition Zone in mm		
	InZ 24 h	InZ 1 Week	InZ 3 Week
*S. aureus*			
a- Group A (n = 30)	16.3 ± 2.1	12.4 ± 1.1	9.4 ± 1.1
b- Group B (n = 30)	21.3 ± 1.4	15.6 ± 1.2	13.3 ± 1.1
c- Group C (n = 30)	19.8 ± 1.9	15.3 ± 1.2	13.2 ± 1.1
d- Group D (n = 30)	25.2 ± 2.2	21.2 ± 1.8	18.3 ± 1.3
e- Group E (n = 30)	10.5 ± 1.6	0	0
ANOVA, F value	16.712	7.172	6.341
ANOVA, *p* value	0.0001	0.003	0.03
Tukey post Hoc	d > e, d > a, d > c, d > b, b > e, b > a, c > e, a > e	d > a, d > c, d > b	d > a, d > b, d >c
*S. mutans*			
a- Group A (n = 30)	18.1 ± 1.9	12.2 ± 1.2	9.3 ± 1.1
b- Group B (n = 30)	19.4 ± 2.1	14.4 ± 1.1	12.1 ± 0.9
c- Group C (n = 30)	20.7 ± 1.5	16.4 ± 1.6	14.4 ± 1.5
d- Group D (n = 30)	26.2 ± 2.6	22.2 ± 2.1	19.1 ± 1.7
e- Group E (n = 30)	9.2 ± 1.2	0	0
ANOVA, F value	17.821	5.328	6.723
ANOVA, *p* value	0.0001	0.04	0.03
Tukey post Hoc	d > e, d > a, d > c, d > b, b > e, c > e, a > e	d > a, d > b, d > c, c > a	d > a, d > b, d >c, b > a, c > a
*C. albicans*			
a- Group A (n = 30)	15.3 ± 1.1	10.2 ± 1.2	8.6 ± 0.9
b- Group B (n = 30)	16.3 ± 1.1	11.2 ± 1.5	9.2 ± 1.3
c- Group C (n = 30)	17.2 ± 1.2	13.4 ± 1.3	10.8 ± 1.1
d- Group D (n = 30)	20.4 ± 2.1	18.2 ± 1.9	16.3 ± 1.3
e- Group E (n = 30)	2.3 ± 1.1	0	0
ANOVA, F value	14.231	6.421	6.723
ANOVA, *p* value	0.0001	0.03	0.03
Tukey post Hoc	d > e, d > a, d > c, d > b, b > e, c > e, a > e	d > a, d > b, d > c, c > a	d > a, d > b, d >c, c > a

Group A—GIC + lyophilized miswak, Group B—GIC + ZOE-AgNPs, Group C—GIC + chlorhexidine diacetate, Group D—GIC + ZOE-AgNPs + Chlorhexidine, Group E—control (glass ionomer alone), InZ—inhibition zone, h—hour, n(a–e)—Number of speciment.

**Table 2:** Comparison of earlier research with restorative materials for antibacterial activity against oral bacteria.

Materials	Concentration	Bacteria	Reference
GIC + CHX	99.5% GIC + 0.5% CHX	*S. mutans*	Kabil et al. [20]
GIC + AgNPs	99.5% GIC + 0.5% AgNPs	*S. mutans*	Paiva et al. [13]
GIC + miswak	GIC + 1.25% miswak	*S. mutans* and *L. casei*	Shahriari et al. [36]
AgNPs + CHX Solution	15 µg/mL AgNPs + 2% CHX	*E. faecalis, K. pneumoniae*, and *C. albicans*	Charannya et al. [25]
GIC + ZOE-AgNPs + CHX	98.5% GIC + 0.5% ZOE-AgNPs + 1% CHX	*S. aureus, S. mutans* and *C. albicans*	This study

**Table 3:** Compressive strength (MPa) of tested specimens.

Study Groups	n	Mean ± SD	ANOVA F Value	ANOVA *p* Value
Group A	10	43.2 ± 3.1	2.326	0.07
Group B	10	44.7 ± 4.8		
Group C	10	43.9 ± 3.6		
Group D	10	45.8 ± 5.4		
Group E	10	42.4 ± 2.7		

Group A—GIC + lyophilized miswak, Group B—GIC + ZOE-AgNPs, Group C—GIC + chlorhexidine diacetate, Group D—GIC + ZOE-AgNPs + Chlorhexidine, Group E—control (glass ionomer alone), SD—standard deviation, ANOVA—analysis of variance.

**Table 4:** Proportion of the Lyophilized miswak, ZOE-AgNPs and Chlorhexidine diacetate added to Conventional GIC.

Group	Abbreviation	Components
Group A	Lyophilized miswak/GIC	(99% GIC with 1% lyophilized miswak, *w*/*w*).
Group B	ZOE-AgNPs/GIC	(99.5% GIC with 0.5% ZOE-AgNPs, *w*/*w*).
Group C	CHX/GIC	(99% GIC with 1% CHX, w/*w*).
Group D	CHX/ZOE-AgNPs/GIC	(98% GIC with 1% CHX, 0.5% ZOE-AgNPs (*w*/*w*).
Group E	GIC	Conventional GIC without any addition.

## Data Availability

Data will be made available on request from corresponding author.

## Sample Availability

Samples of the compounds are not available from the authors.
